# Integrin αIIb-Mediated PI3K/Akt Activation in Platelets

**DOI:** 10.1371/journal.pone.0047356

**Published:** 2012-10-17

**Authors:** Haixia Niu, Xue Chen, Ralph A. Gruppo, Ding Li, Yanhua Wang, Lin Zhang, Kemin Wang, Weiran Chai, Yueping Sun, Zhongren Ding, T. Kent Gartner, Junling Liu

**Affiliations:** 1 Department of Biochemistry and Molecular Cell Biology, Shanghai Key Laboratory of Tumor Microenvironment and Inflammation, Shanghai Jiao Tong University School of Medicine, Shanghai, China; 2 Hematology-Oncology Department, Cincinnati Children’s Hospital, Cincinnati, Ohio, United States of America; 3 Key Laboratory of Molecular Medicine, Ministry of Education, and Department of Biochemistry and Molecular Biology, Fudan University Shanghai Medical College, Shanghai, China; 4 Department of Biological Sciences, University of Memphis, Memphis, Tennessee, United States of America; Lerner Research Institute, United States of America

## Abstract

Integrin αIIbβ3 mediated bidirectional signaling plays a critical role in thrombosis and haemostasis. Signaling mediated by the β3 subunit has been extensively studied, but αIIb mediated signaling has not been characterized. Previously, we reported that platelet granule secretion and TxA2 production induced by αIIb mediated outside-in signaling is negatively regulated by the β3 cytoplasmic domain residues R_724_KEFAKFEEER_734_. In this study, we identified part of the signaling pathway utilized by αIIb mediated outside-in signaling. Platelets from humans and gene deficient mice, and genetically modified CHO cells as well as a variety of kinase inhibitors were used for this work. We found that aggregation of TxA2 production and granule secretion by β3Δ724 human platelets initiated by αIIb mediated outside-in signaling was inhibited by the Src family kinase inhibitor PP2 and the PI3K inhibitor wortmannin, respectively, but not by the MAPK inhibitor U0126. Also, PP2 and wortmannin, and the palmitoylated β3 peptide R_724_KEFAKFEEER_734_, each inhibited the phosphorylation of Akt residue Ser473 and prevented TxA2 production and storage granule secretion. Similarly, Akt phosphorylation in mouse platelets stimulated by the PAR4 agonist peptide AYPGKF was αIIbβ3-dependent, and blocked by PP2, wortmannin and the palmitoylated peptide p-RKEFAKFEEER. Akt was also phosphorylated in response to mAb D3 plus Fg treatment of CHO cells in suspension expressing αIIbβ3-Δ724 or αIIbβ3E_724_AERKFERKFE_734_, but not in cells expressing wild type αIIbβ3. In summary, SFK(s) and PI3K/Akt signaling is utilized by αIIb-mediated outside-in signaling to activate platelets even in the absence of all but 8 membrane proximal residues of the β3 cytoplasmic domain. Our results provide new insight into the signaling pathway used by αIIb-mediated outside-in signaling in platelets.

## Introduction

Integrins are α and β heterodimeric receptors required for numerous essential biological processes [1]. The megakaryocyte- and platelet-specific integrin αIIbβ3 is essential for normal hemostasis [2]. Many integrins on platelets in suspension, including αIIbβ3, are unable to bind their ligands or signal in their low-affinity state [1]. Transformation from the resting state to the active or high-affinity state typically results from integrin-mediated inside-out signaling initiated indirectly by activation of other receptors [1]. This transformation induced by inside-out signaling is controlled by the interaction between the membrane proximal, highly conserved regions of the cytoplasmic domains of the α and β subunits [3]–[6]. Disruption of this interaction by mutation results in the constitutive activation of the affected αIIbβ3 heterodimers expressed in CHO and 293T cells [4], [7], [8]. Agonist-induced physiologic disruption of this interaction appears to be caused by the binding of talin [9], Kindlin [10] or other proteins [1], [11] to the cytoplasmic domain of β3.

The active, oligomerized receptors can also initiate and propagate integrin-mediated outside-in signaling [1]. Binding of a specific ligand to active αIIbβ3 (the gamma chain of Fg does not contain the RGD sequence) induces ‘outside-in’ signaling and results in platelet aggregation, granule secretion and thromboxane A2 (TxA2) production [12]. The contents of the granules, and TxA2 amplify the response to activation, and thereby recruit more platelets to the injury site to participate in thrombus formation [13]. The important roles of β3 in outside-in signaling have been well characterized [14]–[16]. For example, the β3 cytoplasmic domain can bind to a variety of molecules such as c-Src, FAK, and others that propagate outside-in signaling [14]–[22]. Several studies have reported that the calcium-and integrin-binding protein (CIB) can bind to the αIIb cytoplasmic domain and promote β3 mediated outside-in signaling [19]–[21]. More recently, an interesting observation shows that Gα13 directly binds to the β3 cytoplasmic domain and promotes β3 mediated outside-in signaling [14]. And SHARPIN, a α-subunit cytoplasmic domain associated protein, inhibits the recruitment of talin and Kindlin to the integrin, and consequently inhibits the critical switching of β1-integrins from inactive to active conformations [22]. Compared with the deep understanding of β3 subunit-mediated outside-in signaling, little is known about the signaling components utilized by αIIb-mediated outside-in signaling.

In 2005, we reported that αIIb-mediated outside-in signaling is enhanced in platelets of a patient lacking the terminal 39 residues of the β3 cytoplasmic tail (β3Δ724) [23]. This signaling was detected as thromboxane A2 (TxA2) production and granule secretion, and required ligand cross-linking of αIIbβ3 and platelet aggregation. This enhanced outside-in signaling was specifically inhibited by a palmitoylated version of a peptide corresponding to β3 cytoplasmic domain residues R724–R734 (sequence RKEFAKFEEER). Those results demonstrate that αIIb-mediated outside-in signaling resulting in TxA2 production and granule secretion is negatively regulated by RKEFAKFEEER, a sequence of residues in a membrane distal region of the β3 cytoplasmic domain. In this study, we identify some of the signaling molecules that mediate αIIb outside-in signaling.

## Results

### Src Family Kinase (SFK) and PI3K are Key Factors Involved in αIIb-mediated Platelet Activation

In previous work, we showed that the treatment of human β3Δ724 platelets in presence of fibrinogen with LIBS-specific mAb D3 and PT25-2, respectively caused aggregation, TxA2 production and granule secretion [23]. Here, we identify some of the signaling molecules that appear to be involved in these αIIb-mediated responses by using kinase inhibitors. Many studies have demonstrated that Src family kinases [14], [17], [18], PI3K/Akt signaling [24] and MAPK signaling [25] play important roles in platelet activation. But, this direct SFK signaling and subsequent downstream signaling are thought to be dependent of the cytoplasmic domain of β3 [17]. Therefore, the broad spectrum Src family kinase (SFKs) inhibitor PP2, the PI3K inhibitor wortmannin and the MAPK inhibitor U0126 were used in this study.

**Figure 1:**
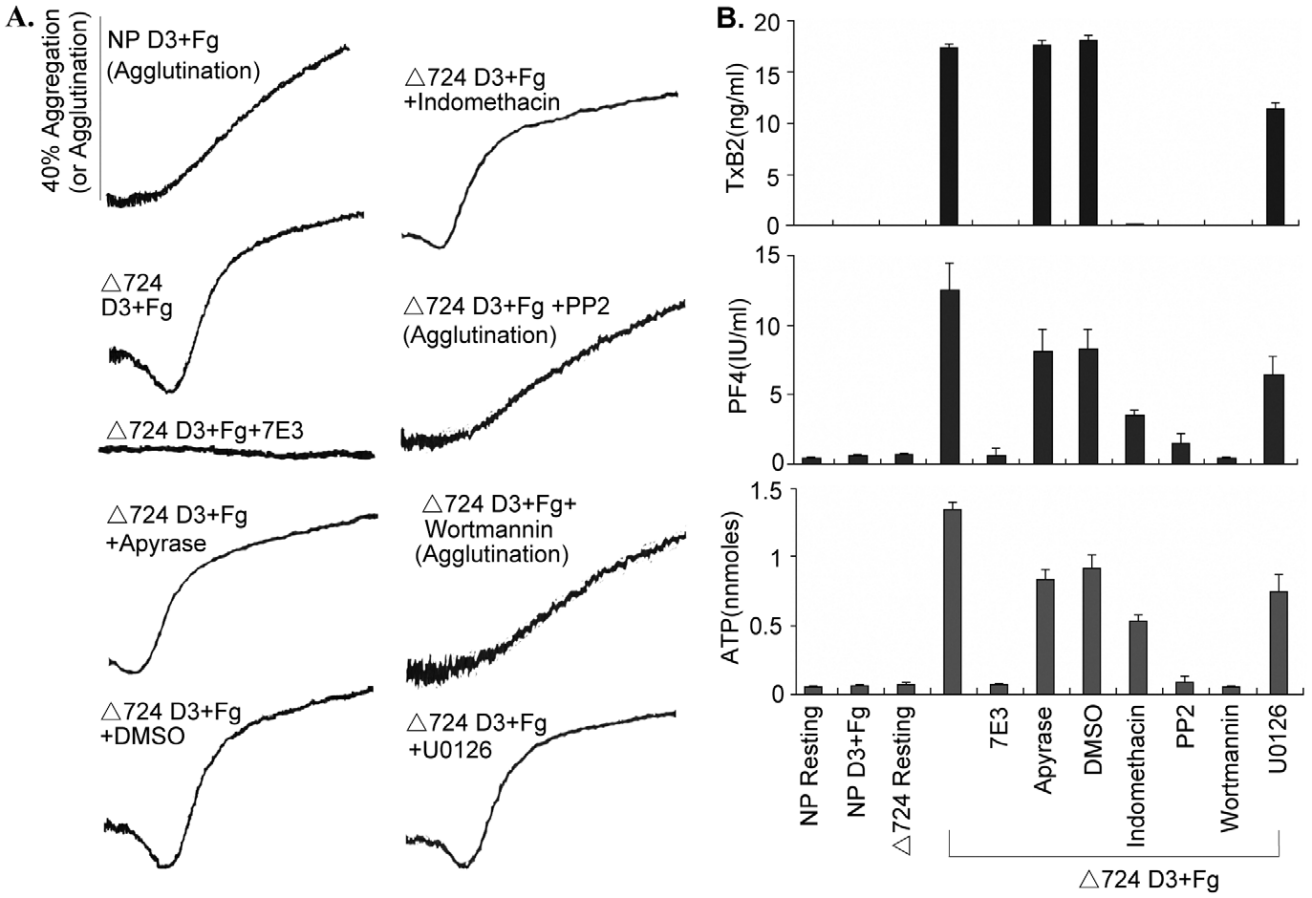
SFK(s) and PI3K propagate αIIb-initiated signaling that elicits platelet activation, TxA2 production and granule secretion. Normal human platelets and β3Δ724 platelets were stimulated by the LIBS-specific monoclonal antibody D3 (30 µg/ml) in the presence of Fg (250 µg/ml) with or without 10 µg/ml of human αIIbβ3 specific monoclonal antibody 7E3, DMSO, 10 U/ml of apyrase, 75 µM of indomethacin, 10 µM of Src family kinase inhibitor PP2, 100 nM of PI3K inhibitor wortmannin, or 10 µM of MAPK inhibitor U0126. (**A**) The agglutination and/or aggregation of normal human platelets (NP) and β3Δ724 human platelets, respectively induced by D3 plus Fg with or without inhibitors treatment. The absence of shape change indicates agglutination, rather than aggregation. (**B**) The TxB2 production and granule secretion of normal human platelets and β3Δ724 platelets induced by D3 plus Fg with or without inhibitor treatment. Each bar represents the mean of quadruplicate determinations. The error bars correspond to the standard deviations of the data.

As we reported previously [23], in contrast to normal platelets, which neither underwent aggregation (they agglutinated, no shape change-Fig. 1A) nor produced TxA2 or secreted the contents of their storage granules, the Δ724 platelets aggregated, produced TxA2, and secreted ATP and PF4 in response to treatment by D3 plus Fg. All these responses were inhibited by the 7E3 (anti human platelet αIIbβ3) (Fig. 1A, B), which confirmed our previous results that Δ724 platelet TxA2 production and secretion induced by D3 plus Fg are aggregation-dependent.

Metabolic inhibitors provided new insight into αIIb outside-in signaling. The Src family kinase inhibitor PP2 and the PI3K inhibitor wortmannin eliminated shape change, and biphasic aggregation and therefore (because those responses require aggregation [23]) TxA2 production and granule secretion by Δ724 platelets in response to D3 plus Fg. These results demonstrate that SFK and PI3K signaling are required for the activation of Δ724 platelets induced by D3 plus Fg. In contrast, the COX inhibitor indomethacin blocked TxA2 production, but had little effect on aggregation and only a moderate effect on granule secretion. Apyrase, an ADP scavenger, had little effect on aggregation, no effect on TxA2 production and a small effect on PF4 secretion. These results revealed that under these conditions, D3 plus Fg induced Δ724 platelet activation is not obviously dependent on signal amplification mediated by TxA2 and ADP. TxA2 production, granule secretion, and aggregation, were slightly diminished in Δ724 platelets treated with MAPK inhibitor U0126 demonstrating that Erk1/2 apparently does not play an essential part in αIIb (D3 plus Fg induced) elicited Δ724 platelet activation (Fig. 1A, B). In summary, despite the fact that Δ724 platelets lack the membrane distal cytoplasmic domain of β3, αIIb elicited platelet aggregation, TxA2 production and granule secretion are SFK and PI3K dependent.

### Akt Phosphorylation in Δ724 Platelets Treated with D3 Plus Fg is Dependent on a SFK(s) and PI3K

**Figure 2:**
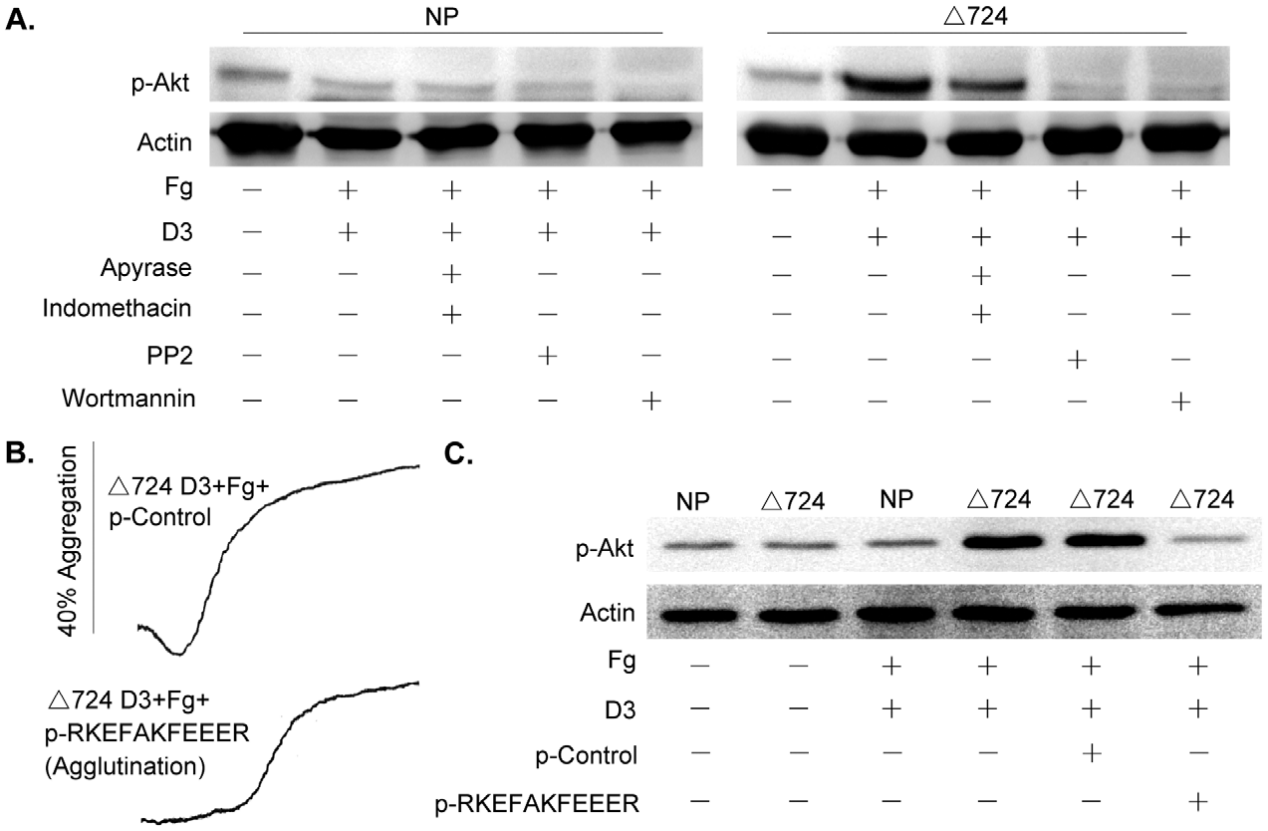
Akt phosphorylation in β3Δ724 platelets elicited by D3 plus Fg is blocked by peptide p-RKEFAKFEEER. Normal and β3Δ724 platelets, respectively were treated with the LIBS-specific monoclonal antibody D3 (30 µg/ml) in the presence of Fg (250 µg/ml), with or without 10 µM of PP2, 100 nM of wortmannin, 10 µM of a p-control peptide, 10 µM of p-RKEFAKFEEER peptide, 75 µM of indomethacin or 10 U/ml of apyrase. Immunoblots of the platelet lysates were treated with anti-phospho-Akt (Ser473) and anti-actin antibodies. (**A**) The Akt Ser473 phosphorylation by normal human platelets and β3Δ724 platelets, respectively induced by D3 plus Fg with or without inhibitors treatment. (**B**) The peptide p-RKEFAKFEEER inhibits the aggregation of β3Δ724 platelets induced by D3 plus Fg. (**C**) The peptide p-RKEFAKFEEER inhibits Akt Ser473 phosphorylation in β3Δ724 platelets aggregating in response to D3 plus Fg. The experiments were repeated for 3 times.

Since wortmannin eliminated αIIb outside-in signaling-dependent aggregation, TxA2 production and granule secretion, activation of the PI3K/Akt pathway in Δ724 platelets treated with D3 plus Fg was investigated. In contrast to the absence of Akt Ser473 phosphorylation in normal platelets, Akt Ser473 was phosphorylated in Δ724 platelets in the presence of Fg in response to D3 (Fig. 2A). Phosphorylation of Akt Ser473 was diminished in the presence of indomethacin plus apyrase indicating that Akt Ser473 phosphorylation may be partially dependent on signal amplification. The Src family kinase inhibitor PP2 and the PI3K inhibitor wortmannin each totally blocked the Akt Ser473 phosphorylation, as well as shape change, aggregation, TxA2 produciton and secretion (Fig. 1). These results demonstrate that D3 plus Fg induced Akt phosphorylation in Δ724 platelets is SFK(s) and PI3K dependent; and, that D3+Fg induced platelet activation apparently is Akt-dependent-though further work is required to establish that point.

Our previous work showed that the palmitoylated peptide RKEFAKFEEER (p-RKEFAKFEEER corresponds to β3 cytoplasmic tail residues 724–734) can block αIIb-mediated platelet aggregation, TxA2 production and granule secretion in response to stimulation by D3 plus Fg without preventing activation of αIIbβ3 [23]. Here, our data reveal that p-RKEFAKFEEER, in contrast to the palmitoylated scrambled control peptide (p-EAERKFERKFE), totally blocks the phosphorylation of Akt Ser473 in Δ724 platelets in response to D3 plus Fg (Fig. 2C), thereby demonstrating that αIIb-mediated activation of PI3K/Akt apparently is also negatively regulated by β3 cytoplasmic domain residues R_724_KEFAKFEEER_734_.

### PAR4 Agonist Peptide (AYPGKF) Induced Akt Phosphorylation in Platelets is αIIbβ3 Dependent and Regulated by the β3 R_724_KEFAKFEEER_734_ Cytoplasmic Domain

**Figure 3:**
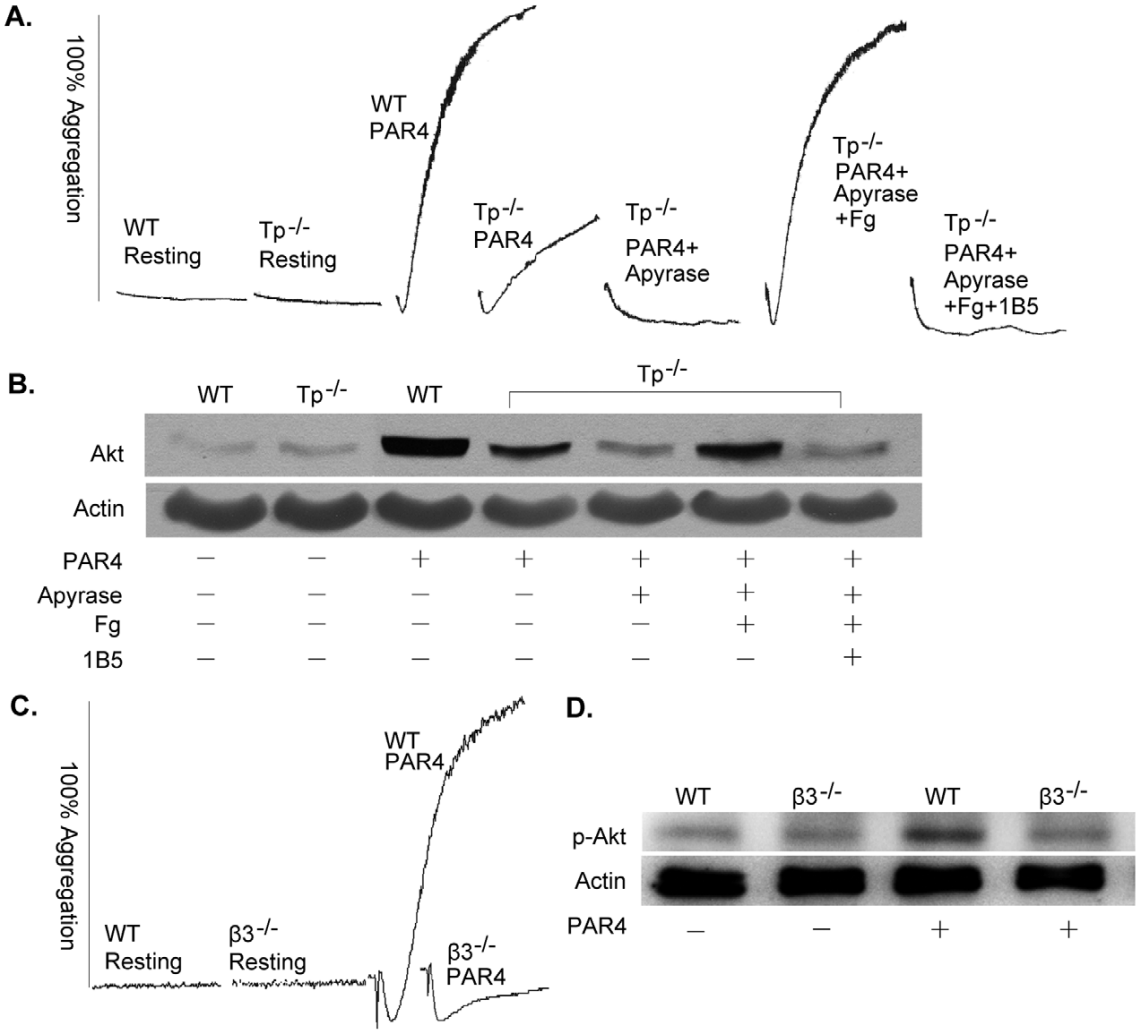
Akt phosphorylation in response to PAR4 stimulation in mouse platelets is secretion- and αIIbβ3-dependent. (**A**) Aggregation of normal and *Tp* deficient mouse washed platelets induced by 160 µM of the PAR4 agonist peptide AYPGKF. Aggregation of *Tp* deficient mouse platelets induced by 160 µM of the PAR4 agonist peptide AYPGKF with and without 10 U/ml of apyrase, ± 250 µg/ml of Fg, ± 10 µg/ml of mAb 1B5. (**B**) Akt Ser473 phosphorylation of normal and *Tp* deficient mouse platelets treated with and without 10 U/ml of apyrase, ± 250 µg/ml of Fg, ± 10 µg/ml of mAb 1B5. (**C**) Aggregation of normal and *β3* deficient mouse washed platelets induced by 160 µM of peptide AYPGKF. (**D**) Akt Ser473 phosphorylation of normal and *β3* deficient mouse washed platelets induced by 160 µM of peptide AYPGKF. The experiments were repeated for three times.

We have shown that αIIbβ3 outside-in signaling induced by γ-thrombin in normal human platelets is negatively regulated by p-RKEFAKFEEER, which implies that αIIb-mediated outside-in signaling is also involved in normal platelet activation [23]. γ-thrombin activates platelets through PAR family receptors on the platelet surface. Here, we characterize αIIb-mediated outside-in signaling in normal platelet activation mediated by PAR family receptors by using mouse platelets. We used the specific PAR4 agonist peptide AYPGKF instead of γ-thrombin because PAR4 is the receptor that mediates signaling in mouse platelets [24], [26]. We used *Tp* deficient mouse platelets to block the amplification of signaling induced by TxA2, and used *β3* deficient mouse platelets to investigate the function of αIIbβ3 in PAR4 mediated platelet activation. As shown in Figure 3A and 3B, wild type mouse platelets aggregated in response to AYPGKF, and Akt was phosphorylated. In contrast, *Tp* ^−/−^ platelets neither aggregated as extensively, nor phosphorylated Akt as extensively as occurred in wild type platelets, in response to stimulation by AYPGKF. Also, apyrase totally inhibited *Tp* ^−/−^ platelet aggregation and Akt phosphorylation induced by AYPGKF. These results implied that PAR4 mediated platelet activation and Akt phosphorylation are amplification dependent. However, exogenous Fg eliminated the inhibitory effects of apyrase. Therefore, α-granule secretion is ADP-dependent, and Akt phosphorylation caused by AYPGKF apparently is aggregation dependent. This conclusion was confirmed by the ability of the mAb 1B5 (prevents Fg binding to mouse αIIbβ3) to prevent platelet aggregation and Akt phosphorylation in platelets stimulated by the PAR4 agonist peptide in the presence of Fg (Fig. 3A, B). Further confirmation of this conclusion was provided by the inability of β*3* deficient platelets (which cannot aggregate) stimulated with AYPGKF to phosphorylate Akt (Fig. 3C, D). These results demonstrate that PAR4 peptide-induced Akt phosphorylation in platelets is αIIbβ3 and aggregation dependent.

**Figure 4:**
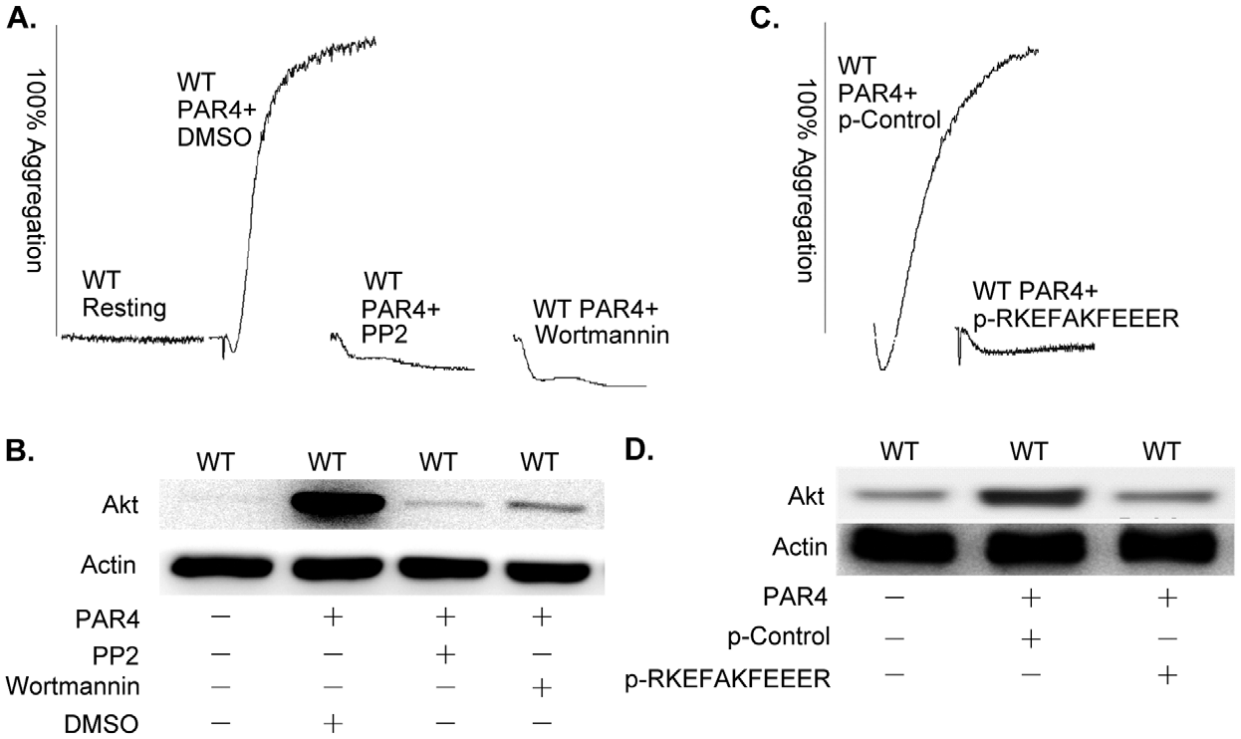
Akt phosphorylation in response to PAR4 stimulation in mouse platelets is SFK and PI3K-dependent, and inhibited by p-RKEFAKFEEER. (**A**) Aggregation of normal mouse washed platelets induced by 160 µM of PAR4 agonist peptide with or without DMSO, 10 µM PP2, or 100 nM of wortmannin. (**B**) Akt Ser473 phosphorylation of normal mouse washed platelets induced by 160 µM of PAR4 agonist peptide with or without DMSO, 10 µM of PP2, or 100 nM of wortmannin. (**C**) Aggregation of normal mouse washed platelets induced by 160 µM of PAR4 agonist peptide with or without 10 µM of p-RKEFAKFEEER or the scrambled p-control peptide. (**D**) Akt Ser473 phosphorylation of normal mouse washed platelets induced by 160 µM of PAR4 agonist peptide with or without 10 µM of p-RKEFAKFEEER or the scrambled p-control peptide. The experiments were repeated for three times.

Further insight into AYPGKF-induced signaling was provided by the use of metabolic inhibitors and peptides. AYPGKF induced aggregation of wild type murine platelets was inhibited by the SFK inhibitor PP2 and the PI3K inhibitor wortmannin (Fig. 4A). Phosphorylation of Akt was completely inhibited by PP2, and diminished 95% by wortmannin (Fig. 4B). The palmitoylated β3 peptide p-RKEFAKFEEER, but not the scrambled p-control peptide (p-EAERKFERKFE) inhibited AYPGKF-induced platelet aggregation (Fig. 4C) and Akt phosphorylation (Fig. 4D). The interpretation of the ability of p-RKEFAKFEEER to inhibit AYPGKF-induced aggregation and Akt phosphorylation is that both of these events are dependent on αIIb-mediated outside-in signaling. Therefore, PAR4 agonist peptide induced activation of normal mouse platelets appears to be caused by αIIb mediated outside-in signaling through SFK and PI3K/Akt signaling pathway.

### The Role of the β3 Cytoplasmic Domain and the R_724_KEFAKFEEER_734_ Sequence in CHO Cell Spreading on Immobilized Fg

**Figure 5:**
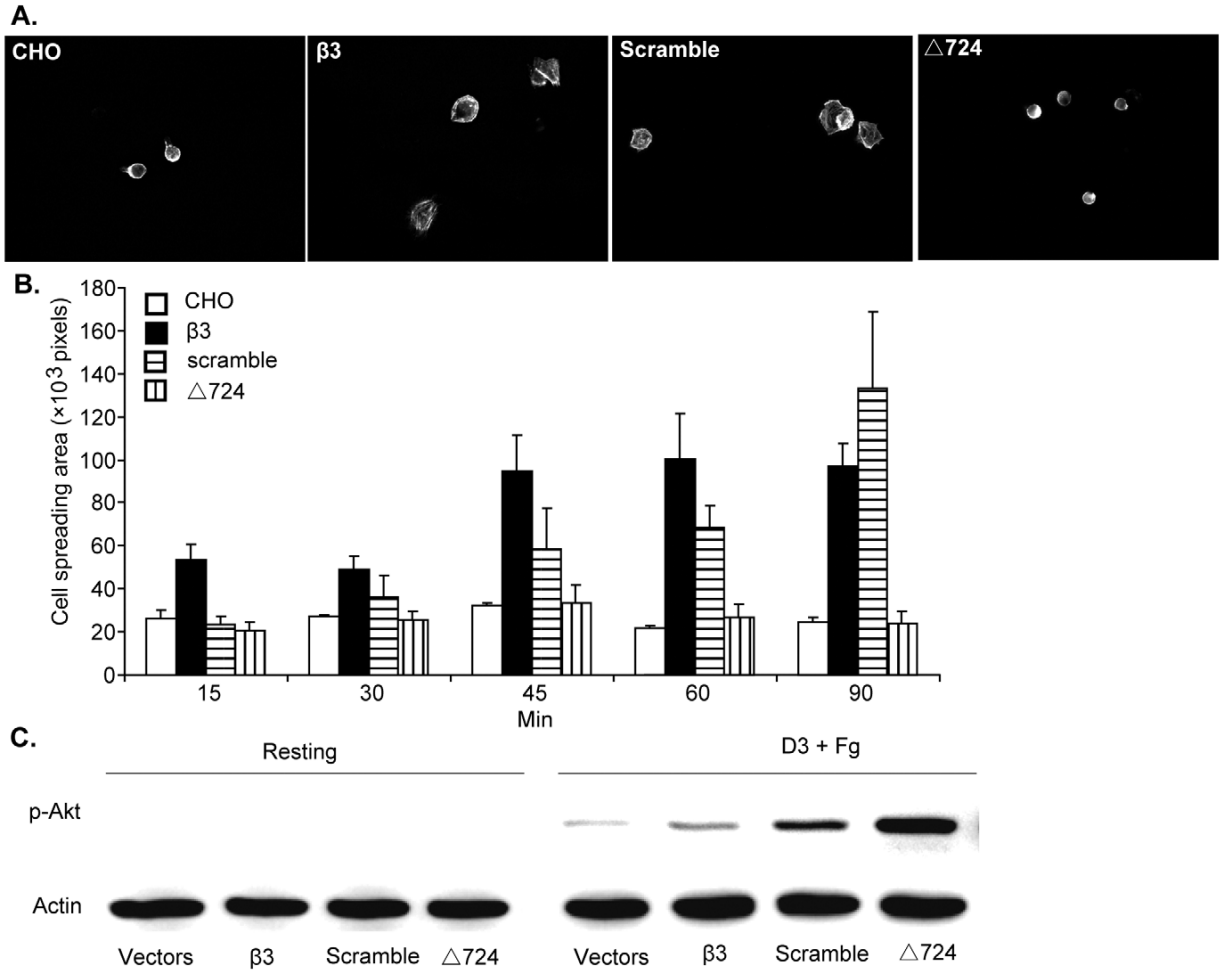
Outside-in signaling mediated by αIIb in CHO cells expressing αIIbβ3. (**A**) Spreading of CHO cells expressing αIIbβ3-WT, αIIbβ3-scramble and αIIbβ3-Δ724, respectively on immobilized Fg for 90minutes. (**B**) Quantification of area (pixel number) in 4 random fields (mean±SE) at all 6 time points. At the 90 minute time point, the size of CHO cells, CHO cells expressing αIIbβ3-WT, αIIbβ3-scramble and αIIbβ3-Δ724, was 24303.43 ± 4851.921 pixels, 97055.33 ± 21284.32 pixels, 133012.3 ± 71539.87 pixels, 24124 ± 11043.93 pixels, respectively. Statistical analysis performed using Student t test. (**C**) Akt Ser473 phosphorylation by CHO cells in suspension expressing αIIbβ3-WT, αIIbβ3-scramble and αIIbβ3-Δ724 induced by 5 µg/ml D3 and 100 µg/ml Fg. These experiments were repeated for three times.

The palmitoylated peptide p-RKEFAKFEEER blocked secretion and Akt phosphorylation in human Δ724 platelets treated with D3 plus Fg, and in wild type murine platelets stimulated by the PAR4 agonist peptide. These results confirm our previous observation that the β3 R_724_KEFAKFEEER_734_ sequence negatively regulates αIIb mediated outside-in signaling. To evaluate this conclusion and investigate the pathway used for αIIb mediated outside-in signaling and regulation using an alternative approach [3], [4], [27], three CHO cell lines stably expressing either human αIIbβ3, αIIbβ3-scramble [a form of αIIbβ3 containing a scrambled version of the R_724_KEFAKFEEER_734_ sequence, (E_724_AERKFERKFE_734_)], or αIIbβ3-Δ724 were constructed. These stable CHO cell lines were evaluated for their ability to spread on immobilized Fg. As with CHO cells transfected with control (no insert) vectors, CHO cells expressing αIIbβ3-Δ724 did not spread on immobilized Fg, while CHO cells expressing normal αIIbβ3 (designated β3) or αIIbβ3-scramble spread fully on immobilized Fg (Fig. 5A). These results demonstrate that the β3 cytoplasmic domain plays a critical role in αIIbβ3-mediated CHO cell spreading, and that the β3 sequence R_724_KEFAKFEEER_734_ is not required for spreading because CHO cells expressing αIIbβ3-scramble (E_724_AERKFERKFE_734_) rather than the normal β3 sequence R_724_KEFAKFEEER_734_, spread normally. Spreading by CHO cells expressing normal αIIbβ3 supports the conclusion that αIIb outside-in signaling is not required for the spreading of CHO cells expressing normal αIIbβ3 on immobilized Fg. Apparently as with platelets [28], [29], truncated β3 expressed in CHO cells is unable to mediate the interaction of αIIbβ3-Δ724 with the cytoskeleton, so those CHO cells are unable to spread on immobilized Fg (Fig. 5, [28]).

#### αIIb mediated PI3K/Akt signaling in CHO cells

Akt phosphorylation was also investigated in all these cell lines in suspension, treated with D3 plus Fg. There was no Akt phosphorylation in the absence of stimulation (resting condition) (Fig. 5B). After stimulation with D3 plus Fg, there was a very low level of Akt phosphorylation in CHO cells expressing normal αIIbβ3. In contrast, Akt phosphorylation was significantly enhanced in CHO cells expressing either αIIbβ3-scramble or αIIbβ3-Δ724. These latter results provide in vivo confirmation, in the absence of p-RKEFAKFEEER, that αIIb-mediated activation of the PI3K/Akt pathway is regulated by β3 cytoplasmic domain sequence R_724_KEFAKFEEER_734_. In view of the results obtained with the modified CHO cells in suspension, it cannot reasonably be argued that the inhibitory results obtained by treating AYPGKF stimulated wild type mouse platelets with p-RKEFAKFEEER were the result of interference of the palmitoylated peptide with β3-mediated inside-out or outside-in signaling. This follows from the fact that replacing the normal β3 sequence R_724_KEFAKFEEER_734_ with a scrambled version of that sequence (E_724_AERKFERKFE_734_) eliminated the ability of the cytoplasmic domain of β3 to prevent Akt phosphorylation.

## Discussion

Bidirectional αIIbβ3 mediated signaling plays an essential role in hemostasis and thrombosis. Contrary to β3-mediated bidirectional signaling, αIIb mediated signaling has not been investigated in detail. Our previous work documented that either of two LIBS-specific mAbs (D3 and PT25-2) plus Fg (but none of the agents alone [23]) can induce human β3Δ724 platelets to undergo shape change, biphasic aggregation (not simply agglutination), secrete the contents of their storage granules and produce TxA2 via αIIb mediated outside-in signaling [23]. Furthermore, those responses were shown to be negatively regulated by the membrane distal β3 cytoplasmic domain residues R_724_KEFAKFEEER_734_ [23]. Here, we identify some signaling molecules activated by this αIIb-mediated outside-in signaling in platelets.

The data presented in Figures 1 and 2 demonstrate that the SFK inhibitor PP2, and PI3K inhibitor wortmannin block αIIb outside-in signaling-induced shape change, platelet aggregation, granule secretion and TXA2 production, thereby demonstrating that a Src family kinase(s) and PI3K/Akt signaling propagate αIIb-mediated outside-in signaling, even in platelets lacking all but eight membrane proximal residues of the β3 cytoplasmic domain. The immunoblotting results in Figure 2A show that αIIb-elicited Akt phosphorylation was inhibited by PP2, and by wortmannin which confirmed the results of preliminary inhibitor screening, and also revealed that the Src family kinase function is upstream of PI3K/Akt signaling as PP2 totally blocked Akt phosphorylation. We have reported that the palmitoylated peptide p-RKEFAKFEEER, the sequence of which corresponds to β3 cytoplasmic domain residues R_724_KEFAKFEEER_734_, negatively regulates αIIb-mediated outside-in signaling, including TxA2 production and secretion [23]. The data in Figure 2C demonstrate that the palmitoylated peptide p-RKEFAKFEEER, but not the palmitoylated control peptide (a scrambled version of RKEFAKFEEER with the sequence EAERKFERKFE) inhibited Akt phosphorylation in Δ724 platelets treated with D3 plus Fg. Therefore, αIIb elicited platelet aggregation, granule secretion and TxA2 production induced by D3 plus Fg treatment of Δ724 platelets is SFK-PI3K/Akt-dependent.

The broad physiologic relevance of αIIb-elicited activation of the PI3K/Akt pathway in platelets is demonstrated by the data in Figures 3 and 4. PAR4 is one of a number of types of thrombin receptors in mouse platelets [30]. The peptide AYPGKF elicits PAR4-induced signaling that causes αIIbβ3 activation [31]. The active αIIbβ3 binds to Fg and initiates amplifying signaling that enhances platelet aggregation. Here, we found that Akt phosphorylation induced in platelets by a low level of AYPGKF is secretion and aggregation dependent. As with Δ724 platelets, p-RKEFAKFEEER, but not the control palmitoylated peptide, inhibited aggregation and Akt phosphorylation induced in wild type platelets by AYPGKF. So, the negative regulation of αIIb-elicited activation of the PI3K/Akt pathway by the β3 cytoplasmic domain residues R_724_KEFAKFEEER_734_ apparently is a normal aspect of the regulation of αIIbβ3-mediated bidirectional signaling in platelets.

In order to confirm the function of β3 cytoplasmic domain residues R_724_KEFAKFEEER_734_ on αIIb-mediated activation of the PI3K/Akt pathway by a means other than the use of p-RKEFAKFEEER, three CHO cell lines expressing integrin αIIbβ3-WT, αIIbβ3-scramble or αIIbβ3-Δ724 were constructed. CHO cells expressing αIIbβ3-Δ724 failed to spread on immobilized Fg demonstrating that the β3 cytoplasmic domain is required for CHO cell spreading on fibrinogen, as with platelets [28], [31]. CHO cells expressing αIIbβ3-WT or αIIbβ3-scramble, spread well. These results confirm that the β3 cytoplasmic domain plays a critical role in platelet spreading, but also provide the new insight that the β3 cytoplasmic domain sequence R_724_KEFAKFEEER_734_ does not participate in CHO cell and probably platelet spreading in a sequence-specific manner. So, integrin αIIbβ3 mediated cell spreading is mainly through β3-mediated outside-in signaling, not αIIb-mediated outside-in signaling. And as stated above, β3 cytoplasmic domain sequence R_724_KEFAKFEEER_734_ is not required for cell spreading-though it isn’t known whether or not deletion of those residues rather than their substitution with EAERKFERKFE, a scrambled version of R_724_KEFAKFEEER_734_ would enable spreading. In this regard, the ability of PMA to cause spreading of CHO cells expressing αIIbβ3-Δ724 (Supplemental Figure S1) supports the conclusion that the β3 CD is required to attach to and/or activate the cytoskeleton of CHO cells, as with platelets [31]. In other words, PMA activation of PKC apparently activates the cytoskeleton thereby enabling the CHO cells expressing αIIbβ3-Δ724 to spread. Activation of the cytoskeleton results in cell spreading even though the truncated β3 presumably cannot directly link to the cytoskeleton. The lack of activation of αIIbβ3-Δ724 is not a barrier to CHO cell spreading on immobilized Fg as resting platelet αIIbβ3 can support cell attachment to Fg [28], [32]–[34].

Regarding D3 plus Fg treatment of CHO cells in suspension, extensive Akt phosphorylation occurred in CHO cells expressing αIIbβ3-Δ724, and substantially less occurred in CHO cells expressing αIIbβ3-scramble in response to stimulation by D3 plus Fg (Fig. 5B), and there was no Akt phosphorylation in CHO cells expressing normal αIIbβ3. These results demonstrate that β3 cytoplasmic domain residues R_724_KEFAKFEEER_734_ negatively regulate αIIb-mediated activation of the PI3K/Akt pathway, and that other β3 cytoplasmic domain residues may also be involved in this regulation.

The potential physiological significance of the αIIb outside-in signaling is that the resulting platelet aggregation presumably could contribute to hemostasis and thrombosis in response to a low, physiologically relevant level of stimulation by an agonist produced/released in response to vascular injury. The αIIb signaling would initiate a positive feedback loop that amplifies the original weak activation induced by the injury, thereby facilitating hemostasis. Assuming the physiological relevance of botrocetin/vwf in-vitro signaling studies [35], αIIb outside-in signaling elicited by vwf/GPIb interaction following arterial plaque rupture or endothelial injury probably would facilitate thrombus formation. This suggestion is supported by the observation that vwf/GPIb interaction initiates signaling that activates αIIbβ3, and the subsequent platelet aggregation enhances TxA2 production, dense granule secretion and Akt phosphorylation [36]. In fact, αIIbβ3 mediated aggregation resulted in about 4 times more TxA2 production than vwf/GPIb mediated agglutination did in the absence of aggregation [37]. Further work is required to test these conjectures.

A number of unanswered questions are raised by our observations. For example, the mechanism of the transfer of signaling information from the cytoplasmic tail of αIIb to a Src family kinase(s) and the PI3K/Akt pathway is unknown. Likewise, the identity of the SFK(s) activated by αIIb signaling is unknown. Also, the mechanism of negative regulation of αIIb-mediated outside-in signaling by key amino acids in β3 R_724_KEFAKFEEER_734_ sequence is unknown. Further work is required to resolve these issues.

In summary, we found that SFK(s) elicited PI3K/Akt signaling is utilized by αIIb-mediated outside-in signaling to activate platelets. Also, our data demonstrate that the negative regulation of αIIb-mediated outside-in signaling by the β3 cytoplasmic domain residues R_724_KEFAKFEEER_734_ prevents activation of the PI3K/Akt pathway. Therefore, our results identified some of the components used by platelets to propagate αIIb-mediated outside-in signaling, an important aspect of platelet function that has not been characterized in detail.

## Materials and Methods

### Ethics Statement

After written, informed consent was obtained, and as part of a routine health exam, extra blood was collected for research purposes from the variant thrombasthenia patient. Approval for the provision of blood for research purposes was obtained from the Institutional Review Board of the Cincinnati Children’s Hospital. After written informed consent, blood was also obtained from a healthy donor to serve as a source of control platelets. The animal research was approved by the Shanghai Jiao Tong University School of Medicine Animal Care and Use Committee (Approve No. SYXK2008-0050).

### Materials

PP2, wortmannin, and U0126 were from Calbiochem (Darmstadt, Germany). Apyrase, PGE1, fibrinogen (Fg) were from Sigma-Aldrich (St. Louis, MO). The anti-phospho-Akt (Ser473), and the anti-actin antibodies were from Cell Signaling Technology (Danvers, MA). The PAR4 agonist peptide, AYPGKF was a generous gift from Dr. Zhongren Ding (Fudan University, Shanghai, China). The monoclonal antibody (mAbs) D3 [38] was a generous gift from Dr. Lisa K. Jennings (University of Tennessee Health Science Center, Memphis, TN). The mAbs 7E3 [39] and 1B5 were generous gifts from Dr. Barry Coller (Rockefeller University, New York, NY). The palmitoylated peptides were synthesized, characterized (by analytical high-performance liquid chromatography and time-of-flight matrix-assisted laser desorption ionization mass spectroscopy) and purified, if necessary, by the Hartwell Center for Bioinformation and Biotechnology (St Jude Children’s Research Hospital, Memphis, TN). The lenti-virus vectors pLVX-IRES-ZsGreen and pLVX-IRES-puromycin were from Clontech (Mountain View, CA).

### Mice

The TxA2 receptor deficient (*Tp* ^−/−^) mice were derived as described [40], wild type littermate siblings were used as controls. Dr. Steven Teitelbaum at Washington University supplied *β3* deficient and littermate control mice.

### Mouse Washed Platelets Preparation and Aggregation

Mice were anesthetized by 1% pentobarbital sodium. Blood was collected from the abdominal aorta of the mice and anticoagulated with White’s anticoagulant (2.94% sodium citrate, 136 mM glucose, pH 6.4) [41]. PRP (platelet-rich plasma) was prepared by differential centrifugation (1000 rpm for 10 min) of blood containing 0.1 µg/ml PGE1 and 1 U/ml apyrase. Washed platelets were prepared by differential centrifugation (2100 rpm for 10 min) of the PRP containing 5 mM EDTA and 1 U/ml apyrase. Platelets were resuspended into modified Tyrode’s Buffer (12 mM NaHCO_3_, 138 mM NaCl, 5.5 mM glucose, 2.9 mM KCl, 2 mM MgCl_2_, 0.42 mM NaH_2_PO_4_, 10 mM HEPES [N-2-hydroxyethylpiperazine-N’-2-ethanesulfonic acid], pH7.4). Aggregation was measured in the Lumi-Aggregometer (Chrono-Log, Havertown, PA) using washed platelets (300 µl) adjusted to approximately 10^6^ platelets / µl with stirring at 1000 rpm.

### Patient

The patient has a variant form of Glanzmann thrombasthenia designated here as β3Δ724 [28]. The patient is a young man with a life-long history of enhanced bruising, mucosal bleeding, and petechiae. He has a normal platelet count but a prolonged bleeding time. The patient contains 2 distinct mutant alleles for β3: an allele that contains a transition (CGA [R] to TGA [nonsense]) mutation that results in a truncated β3 lacking all but the 8 membrane proximal cytoplasmic domain residues. The other allele sustains a deletion of β3 T1181 resulting in a frame shift and a nonsense codon corresponding to amino acid 642. So, the abnormal platelets express only αIIbβ3 complexes containing the truncated β3 subunit, which are otherwise structurally normal. These complexes are expressed at about 40% of the normal level. As a consequence of the truncation, the platelets do not aggregate to physiologic agonists because the truncated β3 subunit cannot mediate inside-out signaling to activate the altered αIIbβ3 complex.

### Human Washed Platelets Preparation and Aggregation

After informed consent was obtained, blood was collected from healthy donors and the variant thrombasthenia patient into empty syringes and then transferred to polypropylene centrifuge tubes containing 100 µl/mL (final concentration) Whites anticoagulant (2.94% sodium citrate, 136 mM glucose, pH 6.4), 0.1 µg/mL PGE1, and 1 U/mL apyrase. Platelet-rich plasma (PRP) was prepared by differential centrifugation. Washed platelets were prepared as described [23]. Aggregation was measured in the Lumi-Aggregometer (Chrono-Log, Havertown, PA) using washed platelets (300 µl) adjusted to approximately 10^6^ platelets / µl with stirring at 1000 rpm.

### Measurement of ATP Secretion

ATP secretion was evaluated using the CHRONO-LUME reagent (CHRONO-LOG Corp., Havertown, PA) as described [12]. After 5 min activation of the stirred platelets (1000 rpm), 10 µl reagent was added directly to each platelet suspension. The luminescence intensity was measured at luminescence setting of ×0.001.

### Measurement of TxA2 Production

TxB2, a stable metabolite of TxA2, was measured as described [12], [42]. TxB2 was measured to indirectly estimate TxA2 production. After a 5 min aggregation period, platelets were removed by centrifugation in the presence of 5 mM EDTA. The supernatant fluid was collected and diluted 1∶50 with the assay buffer supplied in the TxB2 enzyme immunoassay (EIA) kit (Assay Designs, Ann Arbor, MI). TxB2 was measured according the manufacture’s protocol.

### Measurement of PF4 Secretion

PF4 (platelet factor 4) is released from the α-granules by the activated platelets. The supernatant fractions of platelet suspensions were collected and assayed using Asserachrom PF4 quantitative enzyme-linked immunosorbent assay (ELISA) kit (Diagnostica Stago, Parsippany, NJ) as described [43].

### Cloning of Human β3, αIIb and the Mutant Versions of Human β3

pLVX-IRES-Puro-β3 (full-length human β3), pLVX-IRES-Puro-Δ724 (β3 with amino acids 1–724) were generated from human platelet cDNA by polymerase chain reaction (PCR) using a common upstream primer: 5′-CCGCTCGAGGCGGGAGGCGGACGAGAT-3′ (containing Xho I restriction enzyme site), and the following downstream primers: 5′-ACGCTCTAGAGGGAGG GGAAAGGAGGCA-3′ (β3), 5′-CTAGTCTAGACTAGTCGTGGATGGTGATGAGGA-3′ (Δ724) containing Xba I restriction enzyme cutting site. The resultant PCR products were cloned into pLVX-IRES-Puro vector (Clontech). pLVX-IRES-Puro-scramble (the scrambled β3 sequence E_724_AERKFERKFE_734_ was substituted for the normal β3 R_724_KEFAKFEEER_734_ sequence) was generated by overlap extension PCR using flanking primers: 5′-CCGCTCGAGGCGGGAGGCGGACGAGAT-3′ (upstream, containing Xho I restriction enzyme site) and 5′-ACGCTCTAGAGGGAGGGGAAAGGAGGCA-3′ (downstream primer, containing the XbaI restriction enzyme site), and the following overlapping mutagenesis oligonucleotides: 5′-ATCACCATCCACGACGAAGCTGAGCGAAAATTCGAACGCAAATTTGAAGCCAGAGCAAAATGG-3′, and 5′-CCATTTTGCTCTGGCTTCAAATTTGCGTTCG AATTTTCGCTCAGCTTCGTCGTGGATGGTGAT-3′. pLVX-IRES-ZsGreen-αIIb (full length of human αIIb) was generated from human platelet cDNA by PCR using the following primers: 5′-CCGGAATTCAGATGGCCAGAGCTTTGTGT-3′ (upstream primer), and 5′-CTAGTCTAGATGTAGGCTGCACCATCACTC-3′ (downstream primer). The resultant PCR product was cloned into pLVX-IRES-ZsGreen (Clontech).

### Construction of Stable CHO Cell Lines, Spreading of the CHO Cell Lines on Immobilized Fg and Stimulation of Suspensions of the Cell Lines with D3 Plus Fg

Lenti-X cell (Clontech) was used as the lentivirus packaging cell. Lentiviruses were produced by co-transfecting Lenti-X cells with pLVX-IRES-ZsGreen/pLVX-IRES-puro, pspAx2 and pMD2G at 3∶2:1 proportion. The lentiviruses were harvested after transfection, and depletion of the cell debris by centrifugation. CHO cells (Chinese Hamster Ovary cells) were infected with a lentivirus mixture of blank vectors, αIIb and β3, αIIb and β3-scramble, αIIb and β3-Δ724, respectively. Forty eight hours after infection, the infected CHO cells were cultured for two weeks in selective medium containing puromycin (7.5 µg/ml). The viable, GFP positive CHO cells were sorted by FACS. Then the sorted CHO cells were cultured in medium containing puromycin (5 µg/ml) for two weeks. This procedure resulted in the isolation of stable CHO cell lines expressing blank vectors, αIIbβ3, αIIbβ3-scramble, or αIIbβ3-Δ724, respectively. Then, these cell lines were cultured in medium without serum for 12 hrs before further experiments.

The CHO cell lines were allowed to spread for 2 hrs at 37°C on chamber slides coated with 100 µg/ml Fg. Adherent cells were rinsed with PBS and fixed with 4% paraformaldehyde at room temperature. Then the cells were washed with labeling buffer (0.5% BSA, 0.5% Triton X-100 in PBS) and incubated with Rhodamine-phalloidin for 30 min at room temperature for F-actin staining. The slides were washed twice with PBS for the fluorescence microscopy survey. Cell spreading size was quantified using National Institutes of Health (NIH) ImageJ. Statistical significance between groups was determined using a Student t test.

For D3 plus Fg stimulation, the cells were detached from the plates, and resuspended in modified Tyrode solution containing 2 mM Ca^2+^, stimulated with D3 (5 µg/ml), Fg (100 µg/ml) at 37°C for 5 min with stirring at 1000 rpm. Lysates of CHO cells were prepared by adding an equal volume of 2× SDS loading buffer to each cell suspension.

### Western Blot Analysis

Each platelet and CHO cell lysate was boiled at 100°C for 10 min, then resolved on a sodium dodecyl sulfate (SDS), 10% polyacrylamide gel and then transferred to PVDF membrane. Western blots were performed using anti-phospho-Akt (Ser473), or an anti-β-actin antibody at a 1∶1000 dilution, followed by incubation with horseradish peroxidase-conjugated goat anti-rabbit antibody at a 1∶5000 dilution. Blots were developed using Supersignal chemiluminescent substrate (Pierce, Rockford, IL).

## Supporting Information

Figure S1 PMA induced spreading of CHO cells expressing αIIbβ3-WT, αIIbβ3-scramble and αIIbβ3-Δ724. (A) In presence of 100 ng/ml PMA, spreading of CHO cells expressing αIIbβ3-WT, αIIbβ3-scramble and αIIbβ3-Δ724, respectively on immobilized Fg for 90 minutes. (B) Quantification of area (pixel number) in 4 random fields (mean ± SEM) all at 90 minutes. The size of CHO cells, CHO cells expressing αIIbβ3-WT, αIIbβ3-scramble and αIIbβ3-Δ724, was 22519.38±4321.64 pixels, 148891.15±28420.39 pixels, 121496.23±12324.66 pixels, 75023.19±11098.12 pixels, respectively. Statistical analysis performed using Student t test.
(TIF)
