# Supplementary figures and images for: Integrin αIIb-Mediated PI3K/Akt Activation in Platelets
Source: PLoS One. 2012 Oct 17;7(10):e47356. doi: 10.1371/journal.pone.0047356 (PMC3474815; doi:10.1371/journal.pone.0047356)

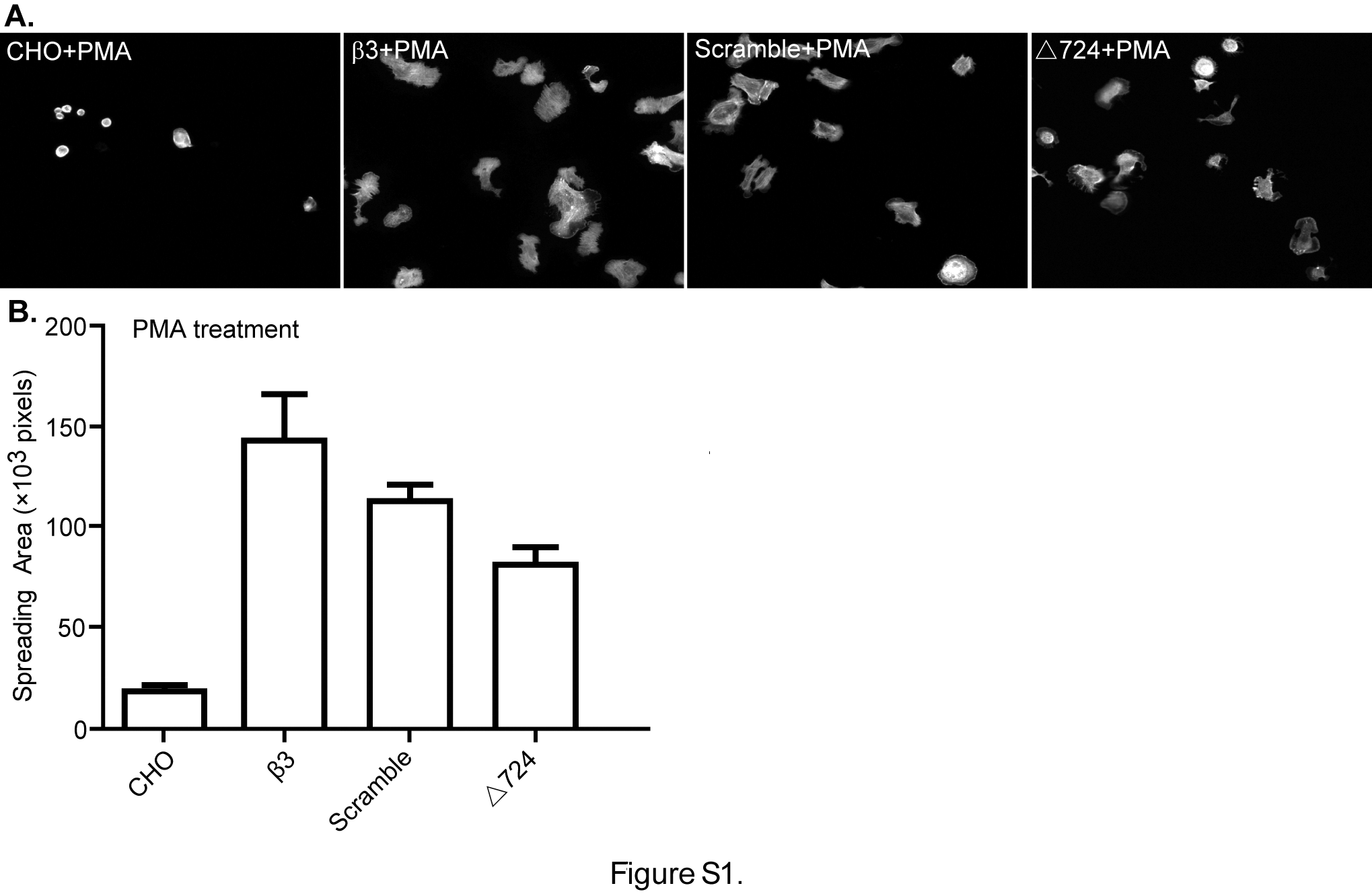

Supplement: Figure S1 — PMA induced spreading of CHO cells expressing αIIbβ3-WT, αIIbβ3-scramble and αIIbβ3-Δ724. (A) In presence of 100 ng/ml PMA, spreading of CHO cells expressing αIIbβ3-WT, αIIbβ3-scramble and αIIbβ3-Δ724, respectively on immobilized Fg for 90 minutes. (B) Quantification of area (pixel number) in 4 random fields (mean ± SEM) all at 90 minutes. The size of CHO cells, CHO cells expressing αIIbβ3-WT, αIIbβ3-scramble and αIIbβ3-Δ724, was 22519.38±4321.64 pixels, 148891.15±28420.39 pixels, 121496.23±12324.66 pixels, 75023.19±11098.12 pixels, respectively. Statistical analysis performed using Student t test. (TIF) [file pone.0047356.s001.tif]
